# Machine learning reveals genetic modifiers of the immune microenvironment of cancer

**DOI:** 10.1016/j.isci.2023.107576

**Published:** 2023-08-09

**Authors:** Bridget Riley-Gillis, Shirng-Wern Tsaih, Emily King, Sabrina Wollenhaupt, Jonas Reeb, Amy R. Peck, Kelsey Wackman, Angela Lemke, Hallgeir Rui, Zoltan Dezso, Michael J. Flister

**Affiliations:** 1 Genomics Research Center, AbbVie Inc, 1 North Waukegan Road, North Chicago, IL 60064, USA; 2 Genomic Sciences and Precision Medicine Center, Medical College of Wisconsin, Milwaukee, WI, USA; 3 Information Research, AbbVie Deutschland GmbH & Co. KG, 67061, Knollstrasse, Ludwigshafen, Germany; 4 Department of Pathology, Medical College of Wisconsin, Milwaukee, WI 53226, USA; 5 Department of Physiology, Medical College of Wisconsin, Milwaukee, WI 53226, USA; 6 Cancer Center, Medical College of Wisconsin, Milwaukee, WI 53226, USA; 7 Genomics Research Center, AbbVie Bay Area, 1000 Gateway Boulevard, South San Francisco, CA 94080, USA

**Keywords:** Microenvironment, Quantitative genetics, Cancer systems biology

## Abstract

Heritability in the immune tumor microenvironment (iTME) has been widely observed yet remains largely uncharacterized. Here, we developed a machine learning approach to map iTME modifiers within loci from genome-wide association studies (GWASs) for breast cancer (BrCa) incidence. A random forest model was trained on a positive set of immune-oncology (I-O) targets, and then used to assign I-O target probability scores to 1,362 candidate genes in linkage disequilibrium with 155 BrCa GWAS loci. Cluster analysis of the most probable candidates revealed two subfamilies of genes related to effector functions and adaptive immune responses, suggesting that iTME modifiers impact multiple aspects of anticancer immunity. Two of the top ranking BrCa candidates, *LSP1* and *TLR1*, were orthogonally validated as iTME modifiers using BrCa patient biopsies and comparative mapping studies, respectively. Collectively, these data demonstrate a robust and flexible framework for functionally fine-mapping GWAS risk loci to identify translatable therapeutic targets.

## Highlights

• Machine learning approach enables functional fine-mapping of breast cancer risk loci
• Prioritized breast cancer I-O targets, including *LSP1* and *TLR1*
• Orthogonal validation in patient tumor biopsies and mouse comparative models
• Flexible framework integrates genotypic, phenotypic, and functional data

## Graphical abstract

**Figure:**
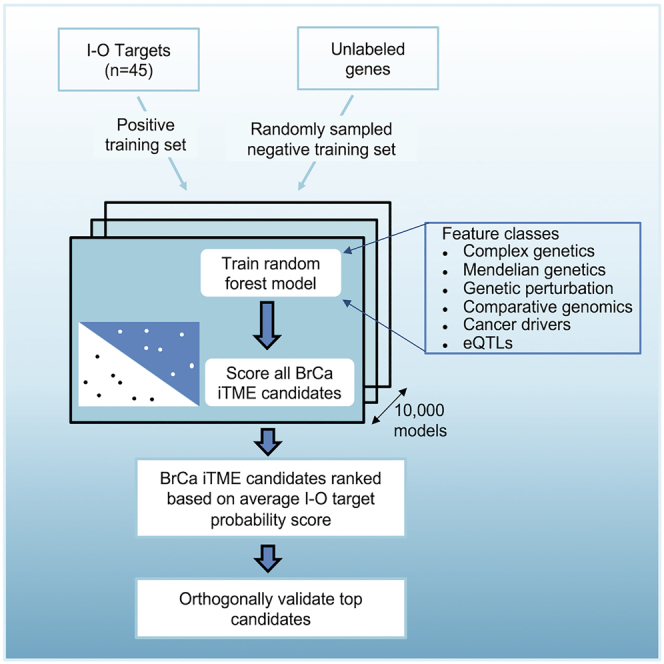

## Introduction

The heritability of breast cancer (BrCa) is estimated to be ∼30%^1^^,^^2^^,^^3^^,^^4^ and germline genetic modifiers have been implicated in most aspects of the disease, including incidence,^5^^,^^6^ age of onset,^7^ distant metastasis,^8^ and survival.^9^^,^^10^^,^^11^ Genome-wide association studies (GWASs) have identified >170 genetic loci to date^12^ and hundreds of genes have been prioritized as candidates by genetic fine-mapping strategies, including functionally validated modifiers of tumor intrinsic phenotypes.^13^ In contrast, germline genetic modifiers of BrCa that function through the tumor microenvironment (TME) remain poorly understood.^5^

The immune system is a key component of the TME, and several recent studies have highlighted germline genetic contributions to antitumor immunity.^5^^,^^14^^,^^15^^,^^16^^,^^17^^,^^18^ A GWAS of The Cancer Genome Atlas (TCGA) pan-cancer cohort revealed heritability (∼15–20%) across 33 traits related to tumor infiltrating leukocytes, including key modulators of antitumor immunity (e.g., IFN, STING, and cytotoxic T-lymphocytes).^15^ Likewise, a separate TCGA pan-cancer analysis found multiple gene-level associations with 16 immune-related traits, including overlapping GWAS candidates for autoimmune diseases.^16^ These data, combined with evidence that similar immune traits predict response to anticancer immunotherapies and overall patient survival,^14^^,^^17^^,^^18^ suggest that germline genetic modifiers of the immune TME (iTME) likely impact cancer incidence, progression, and outcome.

Despite the evidence that heritable variation in antitumor immunity impacts BrCa,^5^ attempts to interrogate germline genetic modifiers of the iTME remain challenging for several reasons. First, well-powered BrCa GWAS (e.g., ∼118,000 cases in BCAC) do not collect iTME phenotypes,^12^^,^^13^ whereas smaller GWAS (e.g., ∼9,000 cases in TCGA) with iTME phenotypes are underpowered for robust genome-wide genetic association.^14^^,^^15^^,^^16^^,^^17^^,^^18^ Second, genome-wide genetic perturbation datasets for functionally annotating candidates have only recently become available^19^^,^^20^ and functional genomics data have yet to be systematically incorporated into fine-mapping strategies for GWAS loci. Finally, in some cases, characterization of iTME modifiers will require comparative analysis using models with a fully intact iTME and yet there is a dearth of genetically diverse syngeneic cancer models for testing iTME modifiers.

The objective of this study was to develop an analytical framework for using human BrCa GWAS as a roadmap for translating iTME modifiers with the greatest therapeutic potential for antitumor immunity. To achieve this objective, a random forest model was trained on a positive set of immune-oncology (I-O) targets using BrCa and immune phenotypes from genetic perturbation studies, comparative genomics, Mendelian genetics, and colocalization with autoimmunity and inflammatory disease risk loci. When applied to 1,362 candidate genes in linkage disequilibrium (LD, *r*^*2*^ > 0.6) with 155 human BrCa GWAS loci, several I-O targets were identified, including *LSP1* and *TLR1*. These data, combined with genomic, transcriptomic, proteomic, and phenotypic evidence, collectively demonstrate that fine-mapping with systematic integration of functional data (i.e., functional fine-mapping) is a powerful approach for translating GWAS risk loci to novel therapeutic targets.

## Results

### Random forest modeling of I-O target probability for breast cancer GWAS candidates

**Figure 1:**
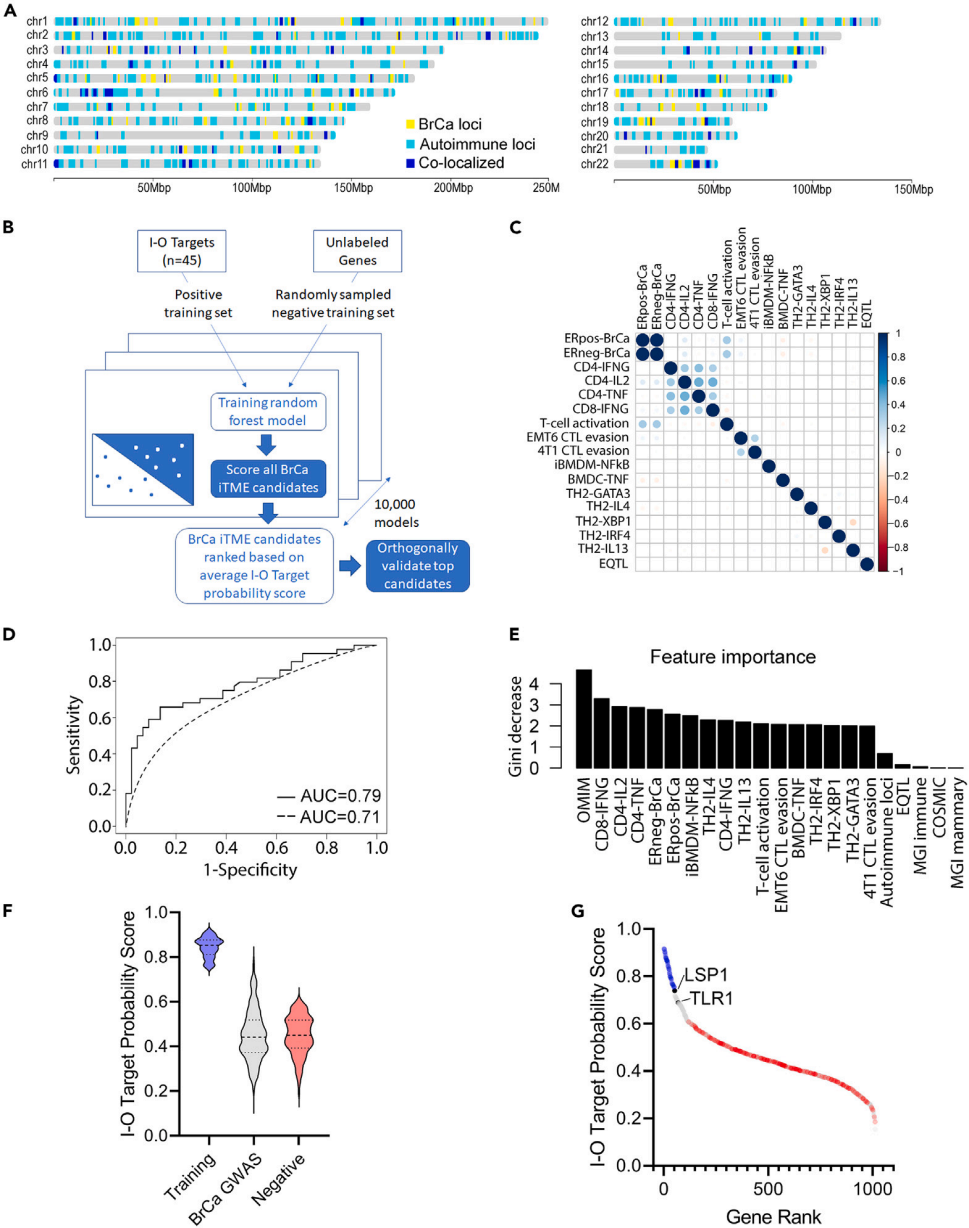
Random forest modeling of I-O target probability for breast cancer GWAS candidates (A) Global alignment of GWAS risk loci for BrCa (n = 155 loci with 452 lead SNPs curated in this study) and autoimmunity (n = 4,076 loci from Open Targets Genetics) revealed significant colocalization (48% of BrCa loci, p < 0.0001). (B) Overview of the I-O target probability predictions using a positive training set of genes with preclinical and clinical evidence as I-O therapies. A size-matched negative set was generated from random sampling without replacement of all other genes and after excluding the positive set. Random forest models were then built (n = 10,000 models) using each of the random negative sets to generate predictions, followed by averaging across the 10,000 models to assign an I-O target probability score to 1,362 candidate genes in linkage disequilibrium (LD, *r*^*2*^ > 0.6) with 155 human BrCa GWAS loci. (C) A correlation matrix of the quantitative variables revealed limited overlap between features. (D) A 10-fold cross validation (CV) was performed using the positive set and each of the negative sets, followed by a receiver operator characteristic (ROC) area under the curve (AUC) analysis that was averaged across all 10,000 random forest models (AUC = 0.71) (Figure 1D, hashed line). Because the randomly sampled negative sets potentially included some unknown I-O targets, a second 10-fold CV was performed using a negative set of 185 genes identified by taking the union of non-essential genes from Hart et al.^21^ and those not expressed in lymph node, bone marrow, thymus, whole blood, lymphocytes, and mammary tissues, based on the Human Protein Atlas and GTEx. Cross-validation with the defined negative set resulted in an AUC of 0.79 (Figure 1D, solid line), indicating that the negative sets likely contain previously undescribed I-O targets. Receiver operating characteristic (ROC) from leave-one-out cross validation using an independent negative set curated from the non-essential gene list and filtered on genes without reported expression in immune or breast cells. The prediction performance was reported as AUC (area under the curve) of 0.79. (E) The relative importance of the 22 features in the Random Forest model as measured by the average decrease in the Gini metric. (F and G) Ranking of I-O target probability scores across the training set (blue), BrCa candidates (gray), and the independent negative set (red). Note the high probability rankings of the BrCa candidates, *LSP1* and *TLR1*.

Recent GWAS in TCGA have demonstrated that germline genetic modifiers of the iTME are prevalent in human cancer, including multiple candidates that are current I-O therapeutics.^14^^,^^15^^,^^16^^,^^17^^,^^18^ Although these GWAS are relatively small and underpowered for candidate gene discovery, the observations suggest that larger cancer GWAS might provide a roadmap for I-O targets with increased translational potential due to the existing association with human malignancy. To this end, colocalization analysis of loci was performed using multiple large publicly available GWAS (N = 809,871 unique individuals) for BrCa (n = 155 loci with 452 lead SNPs curated in this study) and autoimmunity (n = 4,076 loci), which confirmed that heritable immunity is a prevalent component of BrCa risk (48% of BrCa loci, p < 0.0001) (Figure 1A; Tables S1, S2, and S3). However, due to the paucity of immunophenotypic data from these BrCa GWAS, identifying iTME modifiers is not possible without integrating immune phenotypes from other sources, such as Mendelian genetics, comparative genomics, and genetic perturbation studies. The objective of this study was to develop a machine learning approach that would identify the most probable antitumor immune modifiers from BrCa GWAS loci by learning the shared properties of known I-O targets (i.e., the positive set) compared to a negative set of proteins.

We hypothesized that a random forest model that implemented “*easy ensemble*” and *bagging*^22^ would be well-suited to address the challenge of a limited set of true positives (n = 45 curated I-O targets) and a larger set of unknown potential targets (i.e., all other genes). The resulting unbalanced positive-only learning task can be addressed by using negative training sets of equivalent size to the positive set and randomly sampling unique sets that exclude the positive set^23^ (Figure 1B). Random forest models were then built using the averaged predictions over the 10,000 models to assign an I-O target probability score to the 1,362 BrCa candidates in LD (*r*^*2*^ > 0.6) with 155 human BrCa GWAS loci. To capture unique aspects of iTME biology, the random forest model integrated 22 feature sets that included complex genetics (Tables S1, S2, and S3), Mendelian genetics (Table S4), genetic perturbation studies (Tables S5, S6, S7, S8, S9, S10, and S11), comparative genomics (Table S12), cancer drivers (Table S13), and eQTLs (Table S14). A Pearson’s correlation of the model features revealed that there was low covariance between the quantitative variables that were included as features in the random forest model (Figure 1C), suggesting that feature contributions to the model were largely independent.

To assess model performance, a 10-fold cross validation (CV) was performed using the positive set and each of the negative sets, followed by a receiver operator characteristic (ROC) area under the curve (AUC) analysis that was averaged across all 10,000 random forest models (AUC = 0.71) (Figure 1D, hashed line). Because the randomly sampled negative sets potentially included some unknown I-O targets, a second 10-fold CV was performed using a negative set of 185 genes identified by taking the union of non-essential genes from Hart et al.^23^ and those not expressed in lymph node, bone marrow, thymus, whole blood, lymphocytes, and mammary tissues, based on the Human Protein Atlas and GTEx. Cross-validation with the defined negative set resulted in an AUC of 0.79 (Figure 1D, solid line), indicating that the negative sets likely contain previously undescribed I-O targets. The mean decrease in the Gini metric was used to assess the relative importance of the features in the models, which revealed that Mendelian disease feature was the most predictive and followed closely by genetic perturbations and other features (Figure 1E). Combined these features performed expectedly well in predicting the training set (median score = 0.83) compared with the negative training (median score = 0.43) and the BrCa candidates (median score = 0.44) (Figure 1F). Further, the random forest model performed comparably with an elastic-net logistic regression using the 22 feature sets (R = 0.6, p value 10^−92^) (Table S16; Figure S1), suggesting that the performance of the random forest model was not due to overfitting of the limited set of true positives. Collectively, these data demonstrate that a random forest model implementing “*easy ensemble*” and *bagging*^22^ performed well in learning the shared properties of known I-O targets (i.e., the positive set) compared to a negative set of proteins.

Hierarchical clustering of the top ranked BrCa candidates with I-O target probability scores was performed to test whether these plausible iTME modifiers function through similar or distinct biological pathways. STRING analysis was performed on the two major clusters of iTME modifiers, which revealed significant connectivity (p < 1 × 10^−10^) and suggested that highly probable iTME modifiers converge on common biological signaling pathways. Gene ontology (GO) enrichment analysis revealed that Cluster 1 (n = 54 genes) was comprised of more general immune modulators (Table S15A), whereas Cluster 2 (n = 51 genes) was most significantly enriched for modifiers of T cell activation (Table S15B). Since the majority of ICB therapies already focus on the T cell compartment, we chose instead to focus on orthogonal validation of the more broadly acting immune modulators in Cluster 1, which included the highly ranked immune modulators, *LSP1* and *TLR1* (Figure 1G; Table S16). Notably, both candidates are widely associated with BrCa risk^6^^,^^12^^,^^13^^,^^24^^,^^25^^,^^26^^,^^27^ and ranked within the top 25 of genes with the highest I-O target probability scores, yet little is known of the roles of *LSP1* and *TLR1* as genetic modifiers of the iTME. Thus, we sought in subsequent studies to assess the orthogonal evidence of *LSP1* and *TLR1* modifying BrCa risk through the iTME, which would provide further rationale as potential I-O therapeutic targets.

### *LSP1* is an I-O target associated with BrCa risk and outcome

**Figure 2:**
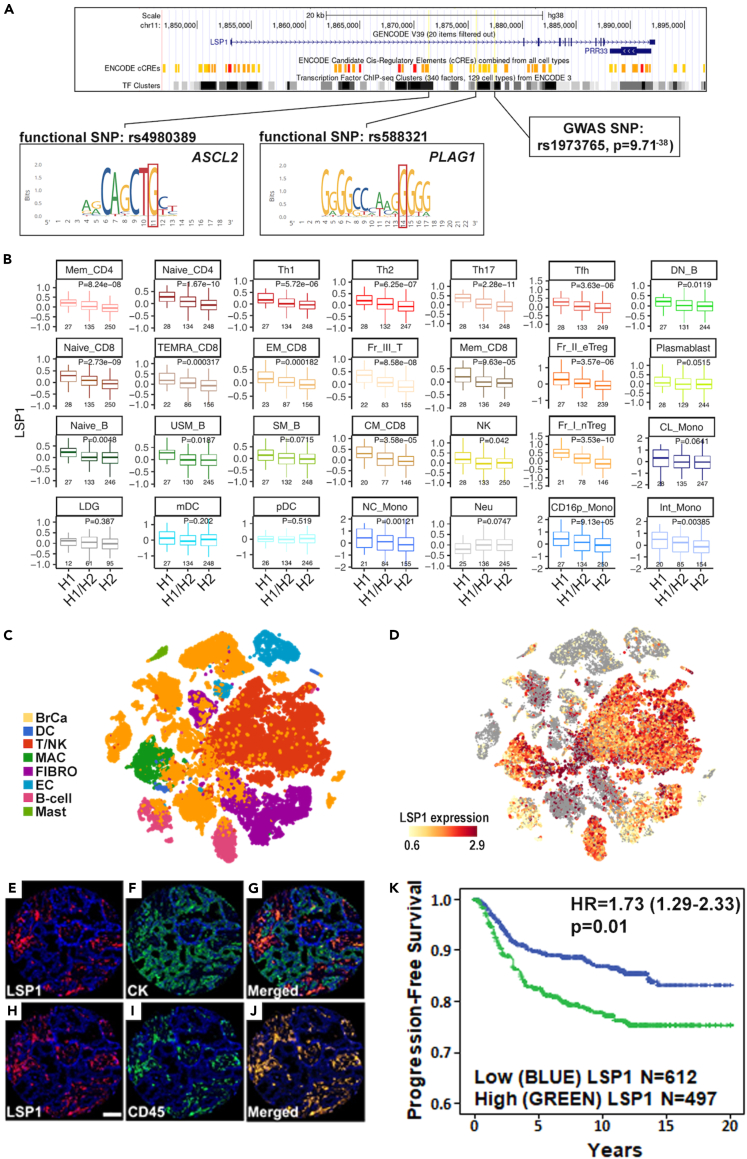
*LSP1* is a germline genetic iTME modifier of BrCa incidence and outcome (A) Annotation of a BrCa-associated *LSP1* locus (rs1973765) in LD (*r*^2^ > 0.6) with multiple fSNPs (e.g., rs4980389 and rs588321) and 17 eSNPs associated with pan-leukocyte expression of LSP1. (B) Haplotype block containing rs1973765 and seven regulatory SNPs is correlated with pan-leukocyte expression of LSP1. Haplotype1 (H1) aligns to rs1973765 T allele and Haplotype2 (H2) aligns to rs1973765 C allele. (C) scRNA-seq analysis of treatment naive BrCa patient biopsies profiled for different cell populations BrCa cells (BrCa), dendritic cells (DC), T-cells and NK cells (T/NK), macrophages (MAC), fibroblasts (FIBRO), endothelial cells (EC), B-cells, and mast cells (MAST). (D) Expression of LSP1 detected by scRNA-seq across the same treatment naive BrCa patient biopsies. (E–J) Quantitative immunofluorescent imaging of LSP1 protein expression colocalized to tumor cells (pan-CK^+^) or infiltrating leukocytes (CD45^+^) in tumors of untreated BrCa patients. Images acquired at 11.6× magnification, panel H scale bar = 100 μm. (K) Progression-free survival analysis based on CD45^+^ leukocyte-specific expression of LSP1 across tumors from 1,109 BrCa patients.

*LSP1* is a negative regulator of leukocyte trafficking and activation,^28^ which has been replicated across multiple GWAS for BrCa incidence^6^^,^^24^^,^^25^^,^^26^^,^^27^ and outcome,^29^^,^^30^ as well as inflammatory and autoimmune diseases,^31^^,^^32^^,^^33^^,^^34^^,^^35^ yet the therapeutic potential of *LSP1* is unknown. Based on the random forest analysis, the probability of *LSP1* as an I-O therapeutic ranked in the top 1% of BrCa GWAS candidates (8^th^ out of 1,362 candidates) (Table S16), suggesting that *LSP1* is an iTME modifier that shares the characteristics of successful I-O targets. Further, seven LD blocks (*r*^2^ > 0.6) localized to *LSP1* have been associated with BrCa incidence or outcome, including 5 LD blocks that correlated with altered LSP1 expression in leukocytes and 29 out of 78 SNPs (37%) within the LD blocks that were predicted to alter a functional motif (Table S17). Notably, 19 of the 29 functional SNPs (fSNPs) (66%) were predicted to disrupt canonical binding sites for transcriptional regulators of immune and inflammatory signaling (Table S17). For example, the most significantly BrCa-associated LD block (led by rs1973765)^26^ contained three fSNPs that were predicted to disrupt *LSP1* regulatory regions, including binding sites for immune regulatory transcription factors, ASCL2 (rs4980389) and PLAG1 (rs588321) (Figure 2A), and eight SNPs correlated with pan-leukocyte expression of LSP1 (Figure 2B). Finally, *LSP1* expression in treatment-naïve tumor specimens from BrCa patients was largely restricted to the infiltrating leukocytes^36^^,^^37^ (Figures 2C and 2D) and high leukocyte-specific expression of *LSP1* correlated with shorter progression-free survival in univariate analysis of BrCa (HR = 1.73, 95% CI 1.29–2.33; p = 0.01) (Figures 2E–2K; Table S18). Combined, these data establish that *LSP1* is a heritable iTME modifier that correlates with BrCa patient survival and closely resembles the characteristics of known I-O targets, which collectively suggest that *LSP1* is a potential I-O therapeutic target with high translational potential.

### Orthogonal validation of *TLR1* as an I-O target using comparative mapping

We recently demonstrated that genetic modifiers in the TME can be mapped using cancer xenograft host strains with distinct genetic backgrounds that differ cancer risk profiles.^38^^,^^39^ Because the xenograft host strain backgrounds vary, whereas the malignant tumor cells do not, any observed changes in tumor progression are due to genetic differences in the nonmalignant TME.^38^^,^^39^ However, a challenge in using human xenografts for TME mapping studies is that the iTME compartment is inaccessible due to the use of immunocompromised xenograft strains. Thus, we developed a strategy for mapping iTME modifiers using syngeneic mouse models with different susceptibility to mammary cancer.

**Figure 3:**
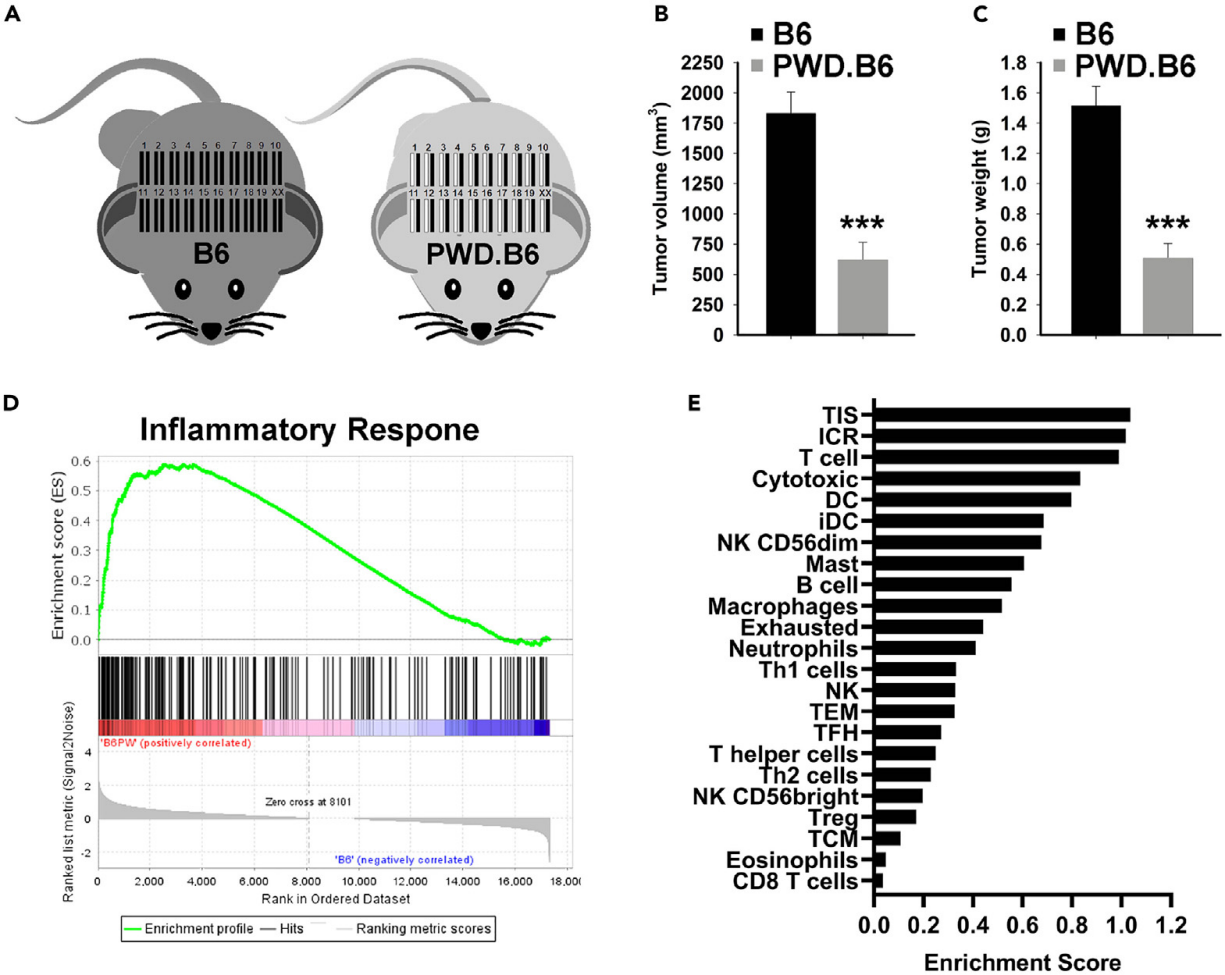
Comparative mapping of iTME modifiers in mouse and human BrCa (A) Schematic representation of syngeneic tumor transplant experiments in homozygous C57BL/6J (B6) mice and PWD/PhJ x C57BL/6J (PWD.B6) F_1_ heterozygous mice. (B and C) E0771.LMB tumor volumes (B) and weights (C) at 17 days post-implantation in B6 homozygous female mice (n = 22) and PWD.B6 F_1_ heterozygous female mice (n = 10). Data are represented as mean ± SEM. ∗∗∗p < 0.001, as determined by Student’s *t* test. (D and E) Enrichment of differentially expressed genes in inflammatory pathways (D) and immune cell infiltrates (E) assessed by GSEA of bulk transcriptomic data from E0771.LMB tumors grown in a B6 and PWD.B6 F_1_ mice (n = 4 per group).

**Figure 4:**
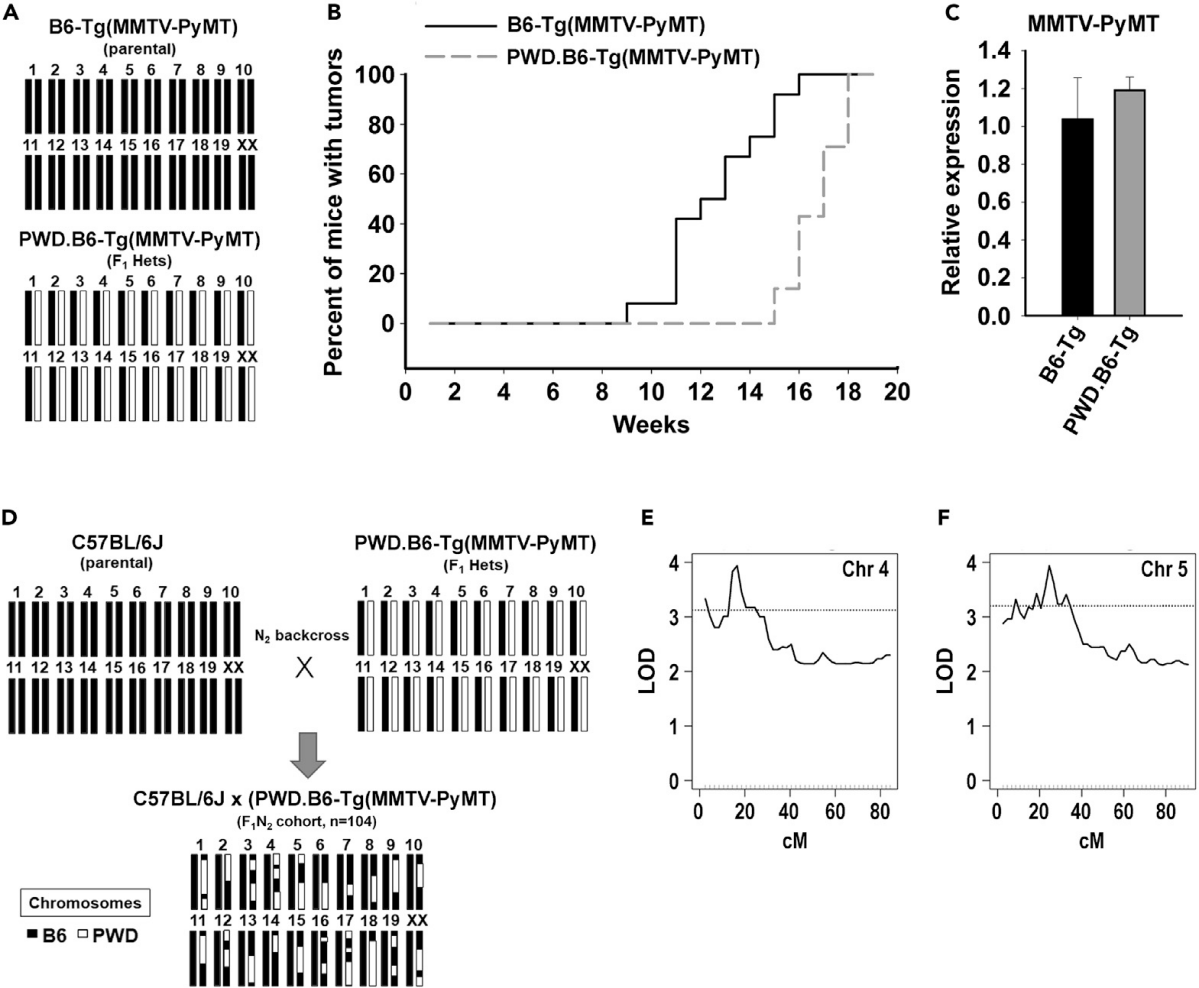
Comparative mapping of iTME loci in the PWD/PhJ mouse background (A) Schematic representation of the genomic backgrounds of homozygous B6-Tg(MMTV-PyMT) mice (black bars) and heterozygous PWD.B6-Tg(MMTV-PyMT) F_1_ mice (white bars). (B) Incidence and latency of mammary tumorigenesis in homozygous B6-Tg(MMTV-PyMT) mice and heterozygous PWD.B6-Tg(MMTV-PyMT) F_1_ mice. (C) Relative expression of MMTV-PyMT in tumors extracted from homozygous B6-Tg(MMTV-PyMT) mice and heterozygous PWD.B6-Tg(MMTV-PyMT) F_1_ mice (n = 4 per group). (D) Schematic representation of the C57BL/6J x (PWD.B6-Tg(MMTV-PyMT)) N_2_ backcross (n = 104). Data are represented as mean ± SEM. (E) Linkage of chromosome 4 with mammary tumor latency when jointly considering the chromosome 5 QTL. (F) Linkage of chromosome 5 with mammary tumor latency when jointly considering the chromosome 4 QTL.

PWD/PhJ is an inbred wild-derived *Mus. m. musculus* strain that is genetically diverse from *Mus. m. domesticus* and therefore well suited for genetic trait mapping with common laboratory strains, such as C57BL/6J.^40^ To assess whether the genetic modifiers observed in the PWD/PhJ genome function through the host TME, the syngeneic C57BL/6J-derived E0771.LMB mammary tumor cell line was implanted into C57BL/6J (B6) homozygous female mice (n = 22) and PWD/PhJ x C57BL/6J (PWD.B6) F_1_ heterozygous female mice (n = 10). Because both host models contain at least one full copy of the C57BL/6J genome, the E0771.LMB cells are syngeneic with the B6 and PWD.B6 host strains (Figure 3A). At 17 days post-implantation, the E0771.LMB tumor burden in PWD.B6 mice (619 ± 147 mm^3^ and 0.5 ± 0.1g) was significantly less than B6 tumors (1,830 ± 188 mm^3^, p < 0.001 and 1.5 ± 0.1g, p < 0.001) (Figures 3B and 3C). Bulk RNAseq analysis of E0771.LMB tumors grown in B6 and PWD.B6 mice (n = 4 per group) revealed 750 differentially expressed genes (DEGs) (Table S19) and gene set enrichment (GSE) suggested increased inflammation (Figure 3D) and leukocyte infiltration (Figure 3E) in PWD.B6 mice. Likewise, known antitumor immunomodulatory pathways were predicted to be highly activated in E0771.LMB tumors implanted in PWD.B6 mice compared with B6: IFNγ (pathway *Z* score = 6.7; p = 1.74^−24^), TNFα (pathway *Z* score = 6.2; p = 8.52^−21^), and IL1B (pathway *Z* score = 4.9; p = 1.29^−22^). To orthogonally test whether the PWD/PhJ and C57BL/6J genomic backgrounds differ in susceptibility to spontaneous mammary tumors driven by the MMTV-PyMT transgene, the latency of mammary tumors in homozygous B6-Tg(MMTV-PyMT) mice were compared with heterozygous PWD.B6-Tg(MMTV-PyMT) F_1_ female mice up to 20 weeks-of-age (Figure 4A). Compared with B6-Tg(MMTV-PyMT), the median tumor latency of PWD.B6-Tg(MMTV-PyMT) F_1_ female mice was significantly delayed by 5 weeks (p < 0.001) (Figure 4B). No differences in MMTV-PyMT transgene expression in the mammary tumors of B6-Tg(MMTV-PyMT) and PWD.B6-Tg(MMTV-PyMT) mice was detected (Figure 4C), indicating that strain-dependent PyMT oncogene expression does not contribute to differences in the mammary tumor risk between B6-Tg(MMTV-PyMT) and PWD.B6-Tg(MMTV-PyMT) mice. Combined with the reduced growth potential of E0771.LMB mammary tumors in PWD.B6 mice, these data suggest that one or more modifiers impact the host iTME to suppress mammary cancer.

**Figure 5:**
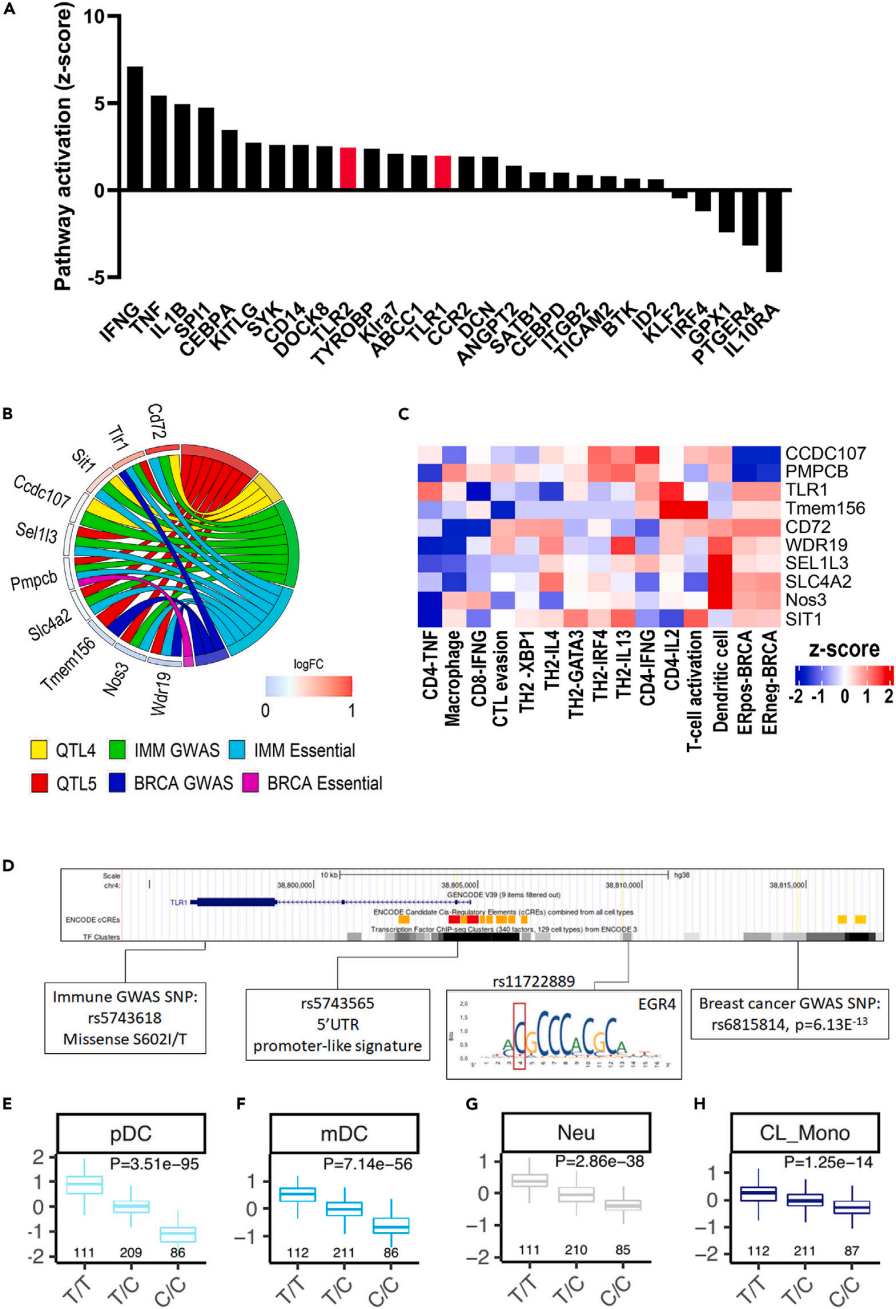
Evidence of *TLR1* as an iTME modifier of BrCa (A) Pathway activation (*Z* score) predicted from bulk transcriptomic analysis of E0771.LMB tumors grown in B6 and PWD.B6 F_1_ mice (n = 4 per group). The highlighted pathways consist of differentially expressed upstream regulators (FDR <0.1) with observable pathway deviations (*Z* score) that passed the accepted statistical threshold (p < 0.01) for the *Upstream Regulator Analysis* function of IPA. Significant activation of the top iTME candidate pathway, Tlr1/2, is highlighted in red. (B) Distribution of intrinsic BrCa phenotypes and extrinsic iTME phenotypes across the candidates overlapping with human GWAS loci. (C) Heatmap of functional fine-mapping scores using CRISPR screening data related to intrinsic BrCa phenotypes and extrinsic iTME phenotypes. (D) Example of a pleiotropic *TLR1* haplotype that colocalizes with risk loci for BrCa (rs6815814) and autoimmunity (rs5743618), as well as multiple fSNPs (rs5743565 and rs11722889) within transcriptional regulatory regions of the *TLR1* locus. (E–H) Examples of eSNP (rs5743565) for TLR1 across multiple human leukocyte cell types, including plasmacytoid dendritic cells (pDC) (E), Myeloid dendritic cells (mDC) (F), neutrophils (Neu) (G), and classical monocytes (H).

Interval mapping of a C57BL/6J x (PWD.B6-Tg(MMTV-PyMT)) N_2_ backcross (n = 104) was used to localize genetic modifier(s) of mammary tumorigenesis (Figure 4D). A genome-wide single locus scan revealed suggestive PWD/PhJ loci that increased mammary tumor latency at chr4 (peak: 34.03 Mb; support interval: 4.68–55.84 Mb) and chr5 (peak: 44.93 Mb; support interval: 15.97–66.36 Mb). When jointly considered, the PWD/PhJ alleles within the chr4 and chr5 loci were significantly associated with increased tumor latency (LOD = 3.9; p = 1 × 10^−4^) (Figures 4E and 4F). The mouse chr4 locus was not syntenic to any reported human BrCa GWAS locus, whereas the chr5 locus was syntenic to one human GWAS locus spanning three candidates (*TLR1*, *WDR19*, and *TMEM156*) that were associated with BrCa risk.^12^^,^^13^ Of the candidates, the *Tlr1* allele had the strongest genotypic and phenotypic evidence, including elevated expression and pathway activation in E0771.LMB tumors implanted in the PWD.B6 genetic background (Figure 5A; Tables S19 and S20), as well as multiple potentially damaging coding variants in the PWD/PhJ background (Tables S21A and S21B). Additionally, *TLR1* had the highest I-O target probability score (0.69) of the candidates, colocalized with autoimmune loci,^41^ and was functionally linked with immune phenotypes^42^^,^^43^^,^^44^^,^^45^ (Figures 5B and 5C). In contrast, no intrinsic BrCa phenotypes were observed for *TLR1* (Figures 5B and 5C). The *TLR1* locus that colocalized with risk loci for BrCa (rs6815814, p = 6.13^−13^) and autoimmunity (rs5743618, p = 4^−114^)^41^ was also in LD (r^2^ > 0.6) with multiple fSNPs predicted to disrupt transcriptional regulatory regions (rs5743565 and rs11722889) (Figure 5D) and multiple eSNPs that correlated with expression across various human leukocyte cell types (Figures 5E–5H). Collectively, these data suggest that *TLR1* is an iTME modifier of BrCa and a molecular target with high translational potential due to the existing association with human BrCa.

## Discussion

Germline genetics contribute to variability in the tumor-infiltrating immune system (referred to as iTME),^5^^,^^14^^,^^15^^,^^16^^,^^17^^,^^18^ which is clinically relevant to disease progression and response to anticancer immunotherapies.^17^^,^^18^ However, most germline genetic modifiers of the iTME remain uncharacterized due to the lack of well-powered GWAS that capture iTME phenotypes. Further compounding the challenges with mapping iTME modifiers is the limited integration of functional and comparative data for iTME-relevant phenotypes into current fine-mapping strategies for GWAS. To overcome these challenges, we developed a machine learning approach that used genomic, transcriptomic, proteomic, and phenotypic evidence to identify the BrCa GWAS candidate genes that most closely resemble features of a positive set of I-O therapeutic targets. This approach prioritized multiple BrCa candidate genes that resembled I-O therapeutics, such as *LSP1* and *TLR1*, which were orthogonally validated by human BrCa disease association and comparative modeling, respectively. Collectively, these data demonstrate that germline genetic iTME modifiers within BrCa GWAS loci are pervasive and fine-mapping these loci with systematic integration of functional data is a powerful approach for translating GWAS risk loci to therapeutic targets.

### Approaches to mapping heritability in the iTME

Although the heritability of germline genetic modifiers of the iTME has long been suspected,^5^ it has only recently been measured using TCGA cohorts that uniquely have both patient genotypes and immune phenotypes inferred from bulk RNAseq.^14^^,^^15^^,^^16^^,^^17^^,^^18^ The estimated heritability of iTME modifiers from these studies approached 20% of variance for some traits,^15^^,^^17^ excluding highly polymorphic regions with much higher estimates of heritability, such as the HLA and KIR regions.^17^ The collective estimates of iTME heritability from TCGA^15^^,^^17^ match the estimated heritability of immune traits in healthy individuals by larger GWAS^32^^,^^46^ and twin studies,^47^ suggesting that the heritability of immunity is consistent in normal and malignant settings. However, despite comparable levels of heritable immunity in normal^46^ and malignant settings,^15^^,^^17^ only a handful of iTME candidates were detected by GWAS in TCGA pan-cancer cohort (∼9,000 patients) as compared with >2,700 modifier loci of immune traits in a much larger cohort of >500,000 healthy individuals.^32^ Together, these observations indicate that GWAS in TCGA is currently underpowered to detect all but the most penetrant iTME modifier loci, and larger cohorts with matched germline and RNA-seq data are needed to replicate and expand the catalog of germline iTME modifiers.^16^ The inadequate power to detect most iTME modifiers by GWAS in TCGA is further compounded by the limitations of deconvoluting cell type-specific signals within bulk transcriptomics data.^48^ Thus, until well-powered cancer patient datasets expand with corresponding immune phenotypes, identifying iTME modifiers is not possible without integrating immune phenotypes from other sources, such as Mendelian genetics, comparative genomics, and genetic perturbation studies.

To our knowledge, this study was the first to develop a machine learning approach to map the most probable antitumor immune modifiers from BrCa GWAS loci by learning the shared properties of known I-O targets (i.e., the positive set) without a labeled set of negative controls. In this setting, the learner only has access to a small set of positive examples and a large set of unlabeled data, because outside the known I-O targets there are many genes with unknown potential as I-O therapeutic targets. This type of machine learning problem is referred to as *Positive Unlabeled* (PU)^49^^,^^50^ and it is compounded by the risk of overfitting when the sizes of the positive and unlabeled sets are unbalanced.^51^ To overcome this problem, we developed a machine learning approach that combines *easy ensemble*^51^ and *bagging*^22^ to balance training sets by randomly sampling negative sets that are size-matched to the positive set.^52^ Random forest models (n = 10,000) were then generated using the positive set and each non-overlapping negative set from the unlabeled data to generate an averaged probability score across all models.^52^ By using known I-O targets as the positive training set, we hypothesized that this approach could be used to prioritize germline genetic modifiers of the iTME with the greatest probability as I-O therapeutic targets. Because this machine learning approach prioritizes candidates associated *a priori* with a disease of interest, the candidates that most closely resemble successful therapeutic targets are also likely to have high translational potential. Finally, this machine learning framework is flexible in that it requires only a positive set for training (i.e., known targets for any disease or phenotype) and can integrate genotypic, phenotypic, and functional data from independent sources to overcome the current limitations of disease association studies.

### BrCa iTME candidates with potential as I-O targets

*LSP1* and *TLR1* were highly ranked within the top 25 genes with the highest I-O probability scores among BrCa candidate genes with orthogonal evidence supporting their role in BrCa. *LSP1* was also distinct from other iTME candidates in that it has been associated with BrCa outcome^29^ in addition to incidence of BrCa^6^^,^^24^^,^^25^^,^^26^^,^^27^ and autoimmunity,^31^^,^^35^^,^^53^^,^^54^ suggesting that *LSP1* is a germline iTME modifier of BrCa incidence and disease progression. Moreover, cell type-specific detection of LSP1 in BrCa tumor specimens revealed that LSP1 expression is predominantly restricted to infiltrating leukocytes and was significantly correlated with better outcome. Based on the observation that *Lsp1*-deficient mice exhibit increased leukocyte motility^28^ and enhanced response to immune checkpoint blockade (ICB),^55^ we hypothesize that *LSP1* is a negative regulator of antitumor immunity. This hypothesis is further supported by the putative role of *LSP1* in neutrophil actin dysfunction (NAD, OMIM 257150), a rare immunological disorder characterized by early onset recurrent infections.^56^^,^^57^^,^^58^ These data, combined with genetic evidence that the *LSP1* locus is associated with altered LSP1 expression in leukocytes and BrCa-specific outcomes, suggest that *LSP1* is a iTME modifier of BrCa incidence and disease progression. *TLR1* was also a notable example of a GWAS candidate for both BrCa^13^^,^^26^ and immune phenotypes,^41^^,^^59^^,^^60^^,^^61^^,^^62^^,^^63^^,^^64^ which were confirmed by comparative mapping and GEMM phenotyping.^65^ TLR1 is a key regulator of innate immune response to DAMPs released by cancer cells^66^ and TLR1/2 agonists have been shown to enhance efficacy of ICB via activation of CTLs.^67^^,^^68^ Combined with the evidence that *LSP1* and *TLR1* both modulate ICB therapies in experimental models,^55^^,^^67^ our data suggesting that *LSP1* and *TLR1* are likely to be patient-relevant targets for modulating antitumor immunity.

### Conclusions

This study systematically mapped germline genetic modifiers of the iTME of human BrCa that most closely resembled the characteristics of known I-O targets, which enabled the discovery of *LSP1* and *TLR1* as iTME modifiers with therapeutic potential. This machine learning framework is flexible in that it required only a positive set for training and could integrate genotypic, phenotypic, and functional data from independent sources to overcome the current limitations of disease association studies. We orthogonally replicated the iTME-specific associations of *LSP1* and *TLR1* to BrCa risk using patient tumor biopsies and comparative mapping, respectively. Importantly, because this machine learning approach prioritizes candidates that were first identified by human BrCa risk association, we postulate that there is greater likelihood that *LSP1* and *TLR1* will be translatable and clinically relevant to BrCa. Combined with our findings, these targets were also independently shown to modify responses to ICB in experimental cancer models.^55^^,^^67^ Thus, we conclude that *LSP1* and *TLR1* are likely to be patient-relevant targets for modulating antitumor immunity. Finally, these data demonstrate a robust and flexible analytical framework for functionally fine-mapping GWAS risk loci to identify the most translatable therapeutic targets for the associated disease.

### Limitations of the study

Despite the evidence of iTME heritability, analytical approaches to identify germline genetic modifiers of the iTME are limited and there are many unresolved questions. One challenge is that iTME candidates are likely to have complex interactions across multiple molecular pathways, cell types, and physiological functions.^5^ Moreover, these interactions might differ across different cancer lineages and subtypes, including differences in the iTME of BrCa subtypes that have recently been reported.^69^ It is also highly plausible that some pleiotropic iTME modifiers impact both BrCa cells and infiltrating leukocytes. Further confounding the ability to characterize iTME candidates is the observation that multiple causative genes can co-segregate at the same GWAS locus.^70^ A single genetic locus could therefore elicit complex biological changes across multiple cell types and the combined effects are ultimately manifested at the phenotypic level. Likewise, seemingly unrelated iTME modifiers that are not connected at the molecular level might interact at the cellular or tissue levels by modifying the density or physiological efficacy of cellular mediators within the tumor. For example, the phenotypic effects of a genetic modifier of infiltrating leukocyte function (e.g., *TLR1*) might be dampened or amplified in a patient that has co-inherited a modifier of leukocyte trafficking (e.g., *LSP1*). To an extent, we demonstrated that potential interactions could be predicted through hierarchical clustering of iTME candidates to identify common biological functions and pathways. However, observation biases potentially exist in this approach due to limitations in the available data. Thus, it is expected that the GOs within iTME modifier subgroups will shift as additional datasets become available that capture different aspects of iTME biology.

The challenges to disentangling the complexities of the host iTME will likely require further development of robust experimental and analytical tools for assessing germline genetic heritability in the iTME. Here, we took the initial step toward developing a machine learning approach to identify the most likely iTME candidates within 1,362 genes in LD (*r*^*2*^ > 0.6) with 155 human BrCa GWAs loci that resemble known I-O targets. To our knowledge, this was the first systematic analysis of BrCa GWAS loci to characterize iTME modifiers using the growing catalog of functional genomics datasets for both oncology and immunology. Intriguingly, this analysis revealed a prevalence of BrCa GWAS candidates (39%) with phenotypic evidence in leukocyte cell types, which also agrees with the significant overlap of 48% (p < 0.0001) of BrCa GWAS risk loci with autoimmune disease loci. Notably, not all candidate phenotypes were observable in *ex vivo* phenotypic screens with isolated cell types, yet perturbation of some of these candidates in the *in vivo* and patient settings revealed strong phenotypic evidence in the iTME. For example, text mining patient data in OMIM was the most predictive feature in the random forest model, which could be attributed to disease phenotypes only observed in patients, such as the association of *LSP1* with the rare neutrophil actin dysfunction disorder (NAD, OMIM 257150). Experimental mapping using the first genetically diverse *in vivo* preclinical models for studying iTME heritability (C57BL/6J and PWD/PhJ), also enabled the independent replication of *TLR1* as a plausible germline genetic modifier of the iTME. While this publication focused on *LSP1* and *TLR1*, many probable BrCA I-O candidates were highly ranked and should be further evaluated. Thus, future studies to expand the repertoire of genotypic, phenotypic, and functional data from preclinical and clinical datasets are likely needed to facilitate the discovery and characterization of additional germline genetic modifiers of the iTME that impact antitumor immunity and response to immunotherapies.

There also remains a need to develop approaches to directly measure the germline genetic contribution to the iTME in patients, which will further enable discovery of additional I-O targets with high translational potential. One possible approach would be to combine germline genotyping with single-cell RNA expression profiling (i.e., scRNA-seq) of dissociated patient tumor biopsies to identify iTME eQTLs, as has been demonstrated elsewhere in circulating leukocytes isolated from healthy individuals.^71^^,^^72^ However, a major drawback of scRNA-seq profiling using dissociated tissues is the inability to capture spatial orientation of profiled cells within the tissue. Spatial transcriptomics was recently developed to enable spatially resolved genome-wide gene expression at the near single-cell level,^73^ yet both scRNA-seq and spatial transcriptomics are cost prohibitive and not scalable to population level studies. Based on recent advances in tissue microarray technologies^74^ and quantitative immunofluorescent imaging,^75^ we propose an alternative framework for iTME QTL mapping at the protein level, using multiplex immunofluorescent assays to correlate cell type-specific protein expression with patient genotypes. The capacity of this approach could be expanded using high density tissue microarrays and quantitative immunofluorescent imaging, which offers highly sensitive and spatially resolved detection of protein expression at the cellular and subcellular levels.

## STAR★Methods

### Key resources table

**Table:** 

REAGENT or RESOURCE	SOURCE	IDENTIFIER
**Critical commercial assays**		

Envision+ HRP-conjugated anti-mouse secondary antibody polymer	Dako	cat# K400311-2
Envision+ HRP-conjugated anti-rabbit secondary antibody polymer	Dako	cat# K4003
Envision+ anti-pan-cytokeratin antibody	Dako	cat# M351501-2
Biotin-conjugated anti-CD45	Biolegend	cat#304004
TRIzol Reagent	ThermoFisher	cat# 15596026
TruSeq RNA library kit	Illumina	cat# S-122-2001
Advantage RT-cDNA kit	Clonetech	cat# 639506
GoTaq qPCR Master Mix	Promega	cat# A6001

**Deposited data**		

FinnGen DF7 GWAS - Malignant neoplasm of breast (controls excluding all cancers)	FinnGen	https://r7.finngen.fi/pheno/C3_BREAST_EXALLC
FinnGen DF7 GWAS - Malignant neoplasm of breast, HER2-negative (controls excluding all cancers)	FinnGen	https://r7.finngen.fi/pheno/C3_BREAST_HER2NEG_EXALLC
FinnGen DF7 GWAS - Malignant neoplasm of breast, HER-positive (controls excluding all cancers)	FinnGen	https://r7.finngen.fi/pheno/C3_BREAST_HERPLUS_EXALLC
FinnGen DF7 GWAS - Malignant neoplasm of breast, HER-negative (controls excluding all cancers)	FinnGen	https://r7.finngen.fi/pheno/C3_BREAST_HERNEG_EXALLC
Michailidou (2017) Nature Breast Cancer GWAS summary statistics	GWAS Catalog	http://ftp.ebi.ac.uk/pub/databases/gwas/summary_statistics/GCST004001-GCST005000/GCST004988/
OpenTargets Autoimmune Disease associations	OpenTargets	https://platform.opentargets.org/disease/EFO_0005140/associations
Online Mendelian Inheritance in Man (retrieval date: 2022-09-22)	OMIM	https://www.omim.org/api
CRISPR knockout screen - LPS induced NFKB activity in an immortalized bone-marrow-derived macrophage (iBMDM)	Covarrubias et al.^42^	https://ars.els-cdn.com/content/image/1-s2.0-S2211124720315308-mmc4.xlsx https://ars.els-cdn.com/content/image/1-s2.0-S2211124720315308-mmc5.xlsx
CRISPR knockout screen - Mouse 4T1 and EMT6 breast carcinoma cell lines propagated in the presence or absence of CTLs	Lawson et al.^43^	https://static-content.springer.com/esm/art%3A10.1038%2Fs41586-020-2746-2/MediaObjects/41586_2020_2746_MOESM6_ESM.zip
CRISPR knockout screen - CD8^+^ T-cell expansion in response to TCR stimulation	Shifrut et al.^44^	https://ars.els-cdn.com/content/image/1-s2.0-S0092867418313333-mmc2.xlsx https://ars.els-cdn.com/content/image/1-s2.0-S0092867418313333-mmc3.xlsx
CRISPR knockout screen - Mouse BMDC	Sheffer et al.^76^	https://static-content.springer.com/esm/art%3A10.1038%2Fs41588-021-00889-w/MediaObjects/41588_2021_889_MOESM3_ESM.xlsx
DepMap CERES essentiality scores, download 4Q19	DepMap	https://depmap.org/portal/
Genome-wide CRISPRi/a screen - Modifiers of IFNg, TNF, or IL2 production	Schmidt et al.^45^	https://www.science.org/doi/suppl/10.1126/science.abj4008/suppl_file/science.abj4008_tables_s1_to_s6.zip
Genome-wide CRISPR screen - Mouse BMDC	Parnas et al.^77^	https://www.cell.com/cms/10.1016/j.cell.2015.06.059/attachment/36e3e4f7-b99d-4613-9129-edd9dd490071/mmc2.xlsx
Genome-wide CRISPR screen - TH2-differentiated T-cells	Henriksson et al.^78^	https://ars.els-cdn.com/content/image/1-s2.0-S0092867418315691-mmc2.zip
MGI Mouse Knockout Phenotypes	Mouse Genome Informatics – The Jackson Laboratory	https://www.informatics.jax.org/
COSMIC Cancer Gene Census	Catalogue of Somatic Mutations in Cancer (COSMIC) database	https://cancer.sanger.ac.uk/cosmic
Leukocyte eQTLs	ImmuNexUT database	https://www.immunexut.org/
scRNAseq from 14 treatment-naïve breast cancer patient	Qian et al.^37^	http://blueprint.lambrechtslab.org

**Experimental models: Organisms/strains**		

Mouse: PWD/PhJ	Jackson Laboratory	RRID:IMSR_JAX:004660
Mouse: C57BL/6J	Jackson Laboratory	RRID:IMSR_JAX:000664
Mouse: B6.FVB-Tg(MMTV-PyVT)634Mul/LellJ	Jackson Laboratory	RRID:IMSR_JAX:022974

**Software and algorithms**		

chromoMap R package	Anand et al.^79^	https://cran.r-project.org/web/packages/chromoMap/vignettes/chromoMap.html
randomForest (v4.6-3)	CRAN	https://cran.r-project.org/web/packages/randomForest/index.html
caret (v6.0-93)	CRAN	https://cran.r-project.org/web/packages/caret/index.html
pROC R package	CRAN	https://cran.r-project.org/web/packages/pROC/index.html
BioTuring package Venice	Le et al.^80^	https://github.com/bioturing/signac
R/qtl	Churchill et al.^81^ Sen et al.^82^	https://rqtl.org/
RSEM (v1.3.0)	Li et al.^83^	“https://github.com/deweylab/RSEM
biomaRt	Durinck et al.^84^ Durinck et al.^85^	https://bioconductor.org/packages/release/bioc/html/biomaRt.html
Bowtie2 (v2.2.8)	Langmead et al.^86^	https://github.com/BenLangmead/bowtie2
DESeq2 (v1.16.11)	Love et al.^87^	https://github.com/mikelove/DESeq2
Ingenuity Pathway Analysis (IPA)	QIAGEN	https://digitalinsights.qiagen.com/products-overview/discovery-insights-portfolio/analysis-and-visualization/qiagen-ipa/
Gene set enrichment analysis (GSEA)	Subramanian et al.^88^	https://www.gsea-msigdb.org/gsea/downloads.jspingen
R package “GSVA” (v 1.34.0)	Hänzelmann et al.^89^	https://bioconductor.org/packages/release/bioc/html/GSVA.html
I2E 6.8[RJ1] [WES2]	Linguamatics / IQVIA Ontology versions used: - Mesh 2022 (2021-12-10) - NCI Thesaurus 21.11e - SNOMED V2021-07-31 - Entrez gene 2022-05-25 - Uniprot 2022-01-19 - Gene-Ontology 2022-05-16	https://www.linguamatics.com/products/i2e

### Resource availability

#### Lead contact

Further information and requests for resources and reagents should be directed to and will be fulfilled by the lead contact (bridget.rileygillis@abbvie.com).

#### Materials availability

This study did not generate new unique reagents.

### Experimental model and study participant details

#### Human breast cancer GWAS

Summary statistics were obtained from the Breast Cancer Association Consortium (BCAC),^26^ UK Biobank,^90^ and FinnGen^91^ (https://www.finngen.fi). The BCAC summary statistics were downloaded from the GWAS catalog (https://www.ebi.ac.uk/gwas/studies/GCST004988).^26^ The UKBB is a large and population-based prospective cohort of approximately 500,000 participants aged 40–69 years recruited between 2006 and 2010 in the United Kingdom.^90^ The UKBB GWAS summary statistics were generated internally defining cases with ICD10 code of C50 (malignant neoplasm of the breast) and controls as individuals without a C50 ICD10 code from the hospital in-patient data. To generate the UKBB GWAS results, a mixed logistic regression model and mixed linear regression model including sex, age, and a genetic relatedness matrix implemented in SAIGEgds^92^ was used to test for single variants associated with the C50 phenotype. FinnGen is a public-private partnership research project combining imputed genotype data generated from newly collected and legacy samples of Finnish biobanks and digital health record data from Finnish health registries. The FinnGen data freeze 7 cohort included 309,154 individuals had been analyzed for over 3,000 endpoints, including incidence of BrCa and autoimmune diseases, and summary statistics can be downloaded here: r7.finngen.fi. In total, the BCAC, FinnGen, and UKBB cohorts represent 152,059 BrCa cases and 657,812 controls of European descent (Tables S1 and S2).

#### Animal models

All animal experiments were performed at the Medical College of Wisconsin (MCW) and all protocols were approved by the Institutional Animal Care and Use Committee of MCW. PWD/PhJ, C57BL/6J, and B6.FVB-Tg(MMTV-PyVT)634Mul/LellJ mice were purchased from Jackson Laboratory and maintained at MCW. The F_1_ and N_1_ strains for phenotyping were generated as follows: PWD/PhJ x C57BL/6J (PWD/C57), C57BL/6J x C57BL/6J (C57/C57), PWD/PhJ x B6.FVB-Tg(MMTV-PyVT)634Mul/LellJ (PWD/C57-PyVT), and C57BL/6J x B6.FVB-Tg(MMTV-PyVT)634Mul/LellJ (C57/C57-PyVT).

#### Mammary tumor transplant model

EO771.LMB cells were orthotopically implanted into PWD/PhJ x C57BL/6J and C57BL/6J x C57BL/6J F_1_ animals and measured as described previously into,^93^ with slight modifications. Briefly, EO771.LMB cells (5 × 10^5^) in 50% Matrigel were implanted into the mammary fat pad (MFP) of age-matched female PWD/PhJ x C57BL/6J F_1_ (n = 10) and C57BL/6J x C57BL/6J N_1_ (n = 22) mice. Tumor volumes were measured weekly by calipers and tumor volumes were calculated according to the formula: volume = *Dd*^2^π/6, where *D* and *d* equal larger and smaller diameters, respectively^94^

### Method details

#### Breast cancer candidate gene mapping

Candidate gene mapping of each set of summary statistics was performed using FUMA,^95^ followed by positional mapping of all SNPs in LD (*r*^2^ > 0.6) with the reported lead SNP and mapping genes to SNPs within 10Kb of the nearest gene using ANNOVAR. Annotated loci from each set of summary statistics were clumped by chromosome and position, then annotated from locus 1 to 155 (Table S2). eQTL mapping was performed for all SNPs using the eQTL Catalogue^96^ DICE,^97^ GTEx (v8),^98^ and eQTLGen.^99^ LD blocks within select loci (e.g., *LSP1* and *TLR1*) were further characterized using Ensembl Variant Effect Predictor (VEP),^100^ ImmuNexUT,^100^ GWAS Catalog,^101^ and RegulomeDB^102^ to annotate SNPs overlapping function motifs (fSNPs).

#### Colocalization of human breast cancer and autoimmunity risk loci

Autoimmunity risk variants were obtained from Open Targets Genetics version 6.^21^ Studies of autoimmune traits were identified using mapped Experimental Factor Ontology terms belonging to the class ‘autoimmune disease’.^103^ Independent lead SNPs from BrCa loci were obtained as described below and are found in Table S3. Locus boundaries shown in the figure, plotted using the chromoMap R package,^79^ were constructed using 250 kb windows on either side of a lead SNP. To test for significant overlap between BrCa loci and autoimmune loci, each of the 155 BrCa loci was randomly assigned a uniformly distributed location on the genome with 10,000 permutations. The distance between the BrCa locus and the nearest autoimmune lead SNP was computed for each set of randomized BrCa locus, and this was used to compute the number of BrCa loci within 500 kb of an autoimmune lead SNP. Results are insensitive to the choice of summary statistic: the mean and median distance, the proportion BrCa loci containing a lead SNP for an autoimmune disease, the proportion of BrCa loci having an autoimmune SNP within varying distances from the locus up to 10 Mb similarly resulted in random distributions that were significantly different from observed values, in most cases without overlap.

#### LSP1 protein expression in human breast cancer tissue biopsy microarrays

A validated multiplexed immunohistochemistry protocol was used to detect LSP protein in tumor-associated leukocytes in 1,109 de-identified cases of invasive BrCa diagnosed at the Jefferson University Hospital from 1988 to 2005 and made available under institutional IRB approved protocols in tissue microarray format (0.6 mm core diameter) as previously described.^73^^,^^74^ In addition to LSP1, multiplex fluorescence staining also included CD45 to detect leukocytes and pan-cytokeratin to detect cancer cells. Immunohistochemistry was performed on an autostainer (Omnis; Agilent/Dako). After antigen retrieval at pH 6, TMA sections were incubated with HRP-conjugated anti-mouse secondary antibody polymer (EnVision+; Dako-Cat# K400311-2); 1:8000 dilution), followed by HRP-conjugated anti-rabbit secondary antibody polymer (EnVision+; Dako-Cat#K4003), and visualized using Cy5-tyramide as substrate. The antigen retrieval step was repeated followed by second round staining for pan-CK using anti-pan-cytokeratin antibody (AE1/AE3 mouse monoclonal, Dako-Cat# M351501-2) followed by HRP-conjugated anti-mouse secondary antibody polymer (EnVision+; Dako-Cat# K400311-2) and visualized by Cy7-tyramide as substrate. CD45 was stained using biotin-conjugated anti-CD45 (Biolegend; cat#304004; 1:50 dilution) and visualized by incubation with Alexa555-labeled streptavidin. DAPI counterstain was used to visualize cell nuclei. Stained slides were digitized at 20x magnification on a slide scanner (3DHistech Pannoramic Flash II) capturing fluorescent images captured in four channels (DAPI, Alexa555, Cy5, ad Cy7. Digitized images were analyzed by Tissue Studio (Definiens) and mean cytoplasmic expression signal for LSP1 immunoreactivity was computed for CD45-positive cells in each tumor core. All immunohistochemistry, slide scanning, and quantitative analyses of digitized images were performed by investigators that were blinded to outcome.

#### RNAseq library preparation and sequencing

Total RNA was collected from EO771.LMB tumors that were xenografted into B6 and PWD.B6 mice (n = 4 per group) isolated using TRIzol Reagent (ThermoFisher), poly-A purified, transcribed, and chemically fragmented using Illumina’s TruSeq RNA library kit using the manufacturer’s protocol. Individual libraries were prepared for each sample, indexed for multiplexing, and then sequenced on an Illumina HiSeq 2500 (Illumina, Inc., San Diego, CA).

#### Spontaneous mammary tumor model

The F_1_ generations of the PWD/C57-PyVT and C57/C57-PyVT crosses were genotyped for the PyVT transgene using an established protocol from the Jackson Laboratory. Beginning at 6 weeks-of-age, the transgene-positive PWD/C57-PyVT and C57/C57-PyVT female mice were palpated weekly for mammary tumors, and the initial date of detection and anatomical location were recorded. Prior to tissue collection, tumor-positive mice were then aged for 40 days post-diagnosis to permit development of metastases. To localize the mammary tumor modifiers in the PWD/PhJ and C57BL/6J genomic backgrounds, a [C57BL/6J x (PWD/PhJ x B6.FVB-Tg(MMTV-PyVT)634Mul/LellJ)]N_2_ backcross (n = 104) was performed. Genome-wide genotyping was performed using 3,753 informative markers from the MUGA array.^104^

### Quantification and statistical analysis

#### Random forest modeling of I-O target probability

The random forest models and predictions were performed using the “randomForest (v4.6-3)” and “caret (v6.0-93)” libraries in R statistical software. The number of trees in each model was fixed at 1000, as any increase in trees above this value did not yield any significant changes in the prediction probability. The tuneRF function in the random forest package was used to determine the optimal number of variables (“mtry”) sampled at each split. The stepfactor was set at 0.01 and the “improve” parameter at 0.01 value. A positive training set of 45 preclinical and clinical I-O therapeutic targets were identified from the literature.^105^ To balance the training set for the model, size-matched negative training sets were generated by random sampling without replacement of genes and excluding the positive training set. Genes with more than 6 missing values were omitted from the predictions. A total of 10,000 random forest models using each of the random negative sets were trained and predictions were made based on each model, followed by averaging the predictions over 10,000 models to assign the IO target probability score to each gene. Two methods were used to cross-validate the random forest model. In the first approach, a 10-fold cross-validation (CV) was performed using the positive set (n = 45) and a negative set of 185 genes identified by taking the union of non-essential genes from Hart et al.^23^ and those not expressed in lymph node, bone marrow, thymus, whole blood, lymphocytes, and mammary tissues, based on the Human Protein Atlas and GTEx. This resulted in a CV of AUC = 0.79. In the second approach, a 10-fold cross-validation (CV) was performed for each of the 10,000 random forest models that were generated using the positive set (n = 45) and the 10,000 size-matched and randomly selected negative sets, followed by averaging of the 10,000 AUCs. This resulted in a CV of AUC = 0.71. The slight drop in the AUC of the second approach is likely due to the random sampling of negative sets that were used for the 10,000 random forest models, which very likely included some models built from negative sets that included unknown I-O targets. The random forest analysis was further benchmarked against an elastic-net logistic regression model with L1 and L2 regularization to minimize overfitting. For each elastic-net model, the alpha and lambda parameters were tuned to maximize accuracy based on a 10-fold cross-validation using the “train” function from the “caret” R package. Identical to the random forest analysis, the positive set (n = 45) was sized-matched with randomly-sampled negative sets without replacement of genes and excluding the positive training set. A total of 10,000 elastic-net models using each of the random negative sets were trained and predictions were made based on each model, followed by averaging the predictions over 10,000 models to assign the IO target probability score to each gene. The elastic-net model was then cross-validated using the same two approaches that were applied to the random forest analysis: (a) the positive set (n = 45) and a fixed negative set of genes (n = 185) that yielded an AUC of 0.77 and (b) the positive (n = 45) and the 10,000 randomly sampled negative sets (n = 45) that yielded an average AUC of 0.69. Finally, the scores from the random forest and the elastic net-analyses were compared by Pearson's correlation, which revealed significant agreement between the approaches (R = 0.6, p-value 10^-92^) (Table S16; Figure S1). Combined, the results of the two cross-validation approaches suggest that the probability scores assigned to the BrCa candidate genes are accurate and not a result of overfitting.

Twenty-two features were included in the random forest model:

• *OMIM Mendelian genetics* – The Online Mendelian Inheritance in Man® (OMIM®) database (https://omim.org/) was mined using Natural Language Processing (NLP) and ontology-based Text and Data Mining (TDM) to extract immunology and cancer phenotype relevant concepts. These data were then converted to a numerical matrix that was compatible for random forest modeling. The full OMIM was obtained via FTP (Morbid Map, Gene Map) and API (full records) respectively (retrieval date: 2022-09-22) and pre-processed with Python for TDM indexing. The information was mined from specific sections (depending on the concept) in the disease and gene monographs, as well as clinical synopses using IQVIA/Linguamatics I2E platform.^106^ We further utilized internally built knowledge extraction pipelines and disambiguation routines.^107^ TDM queries were focused on paragraphs with clinical / human focus. Linguistic context (sentiment analysis) was used to filter out hits related to normal phenotype (“normal leukocyte count”) or absence of abnormality (“no autoantibodies detected”). TDM identified the most specific concept in the text as hit. In post-processing, ontological hierarchies are considered, and matches propagated to higher level concepts to balance the curator-dependent level of granularity found in OMIM text. For TDM, a list of concepts for normal and pathological immunity were mapped, including those related to immune cells, immunoglobulin types, autoantibodies, anatomical parts of the immune system like spleen, autoimmunity, immunodeficiency, recurrent infection, hypersensitivity, atopy, inflammatory reaction, immune system diseases, leukocyte disorders, and lymphatic diseases (see column headers in Table S4 for full list of concepts). Likewise, TDM was used to generate a list of concepts for cancer relevant processes (e.g., proliferation, apoptosis) for solid and hematological tumors (Table S4). The concepts were mapped to public standard life science ontologies,^93^^,^^94^^,^^108^^,^^109^ and internally enriched with additional synonyms and custom-built vocabularies for concepts that could not be mapped to external standards. Disease concept associations resulting from mining the disease monographs and clinical synopses were turned into indirect Gene – I-O concept associations using enriched OMIM Gene – Disease (G-D) relationships. These relationships use Morbid Map data (6862 G-D relationships) as a basis and are enriched with TDM (5895 additional G-D associations) by mining ‘Molecular Basis’ in clinical synopses and linguistic relationship mining in relevant disease paragraphs. TDM enrichment is crucial as, for example, the association between LSP1 and Neutrophil Actin Dysfunction is only described in the text but not covered in Morbid Map and no indirect LSP1 and I-O associations could have been derived. Finally, all individual direct or indirect gene I-O concept associations were summarized into the binary per gene I-O association matrix (see Table S4). The final OMIM input feature to the random forest model is the sum of concept hits for each gene.
• *Genetic perturbation data* – Published genome-wide CRISPR knockout (KO) screens various BrCa cells^19^ and immune cell phenotypes^42^^,^^43^^,^^44^^,^^76^ were curated from the literature. For mouse screens, the mouse genes with human orthologues were converted to the human gene symbols using Ensembl Biomart.^85^^,^^110^ All screening data are provided in the supplement in the original format and standardized format prior to random forest analysis, as follows: z score = (value – mean) / (standard deviation).
  ○ ER+ ER- BrCa DepMap: Genome-wide gene essentiality scores across 40 human BrCa lines (n=10 ER^+^, n=30 ER^-^) in the DepMap (downloaded 4Q19). Essentiality scores were calculated by the DepMap consortium using CERES^111^ and averaged across ER^+^ and ER^-^ negative BrCa subtypes (Tables S5A–S5C).
  ○ CD4 CD8 Schmidt: Genome-wide CRISPRi/a screens of the modifiers of IFNg, TNF, or IL2 production in TCR-stimulated human T-cells.^45^ CRISPR data were analyzed by the authors using MAGeCK version 0.5.9.2. Table column headers are defined as: (A) Gene = Gene name; (B) Screen_Version = Primary CRISPRa/CRISPRi in CD4/CD8 T cells or supplementary screens in CD4 T cells; (C) CRISPRa_or_i = CRISPRa or CRISPRi screen; (D) CD4_or_CD8 = Screen in CD4 or CD8 T cells; (E) Cytokine = Cytokine screened for; (F) LFC = sgRNA median of Log2FoldChange of High/Low sorting bin counts; (G) zscore = Z-Score of LFC values (CRISPRa screens) or -1∗LFC (CRISPRi screens), such that positive regulators will have positive Z-Scores and vice versa; (H) FDR = Gene FDR (MAGeCK RRA test); (I) Hit = Hit criteria met (FDR < 0.05 & absolute LFC > 0.5) ; (J) Hit_Type = Positive or Negative regulator (Tables S6A and S6B).
  ○ Tcell Shifrut: Genome-wide CRISPR KO screen of CD8^+^ T-cell expansion in response to TCR stimulation.^44^ Significant guide enrichment and depletion was analyzed using MAGeCK and alpha-robust rank aggregation (RRA) to obtain gene-level scores, p-value, FDR, and log fold change (lfc) (Tables S7A and S7B).
  ○ CTL evasion: DrugZ output summary from genome-wide CRISPR KO screens performed in mouse 4T1 and EMT6 breast carcinoma cell lines propagated in the presence or absence of CTLs.^43^ DrugZ v1 was used to quantitate the fitness effect of gene perturbation under CTL selection. Positive NormZ score indicates gene perturbation results in resistance, whereas negative NormZ score indicates enhanced sensitivity. (Tables S8A and S8B).
  ○ iBMDM-NFKB: Genome-wide CRISPR KO screen of LPS induced NFKB activity in an immortalized bone-marrow-derived macrophage (iBMDM)-Cas9 with an NF-KB-GFP transgenic reporter.^42^ Fold enrichment in LPS-stimulated iBMDM cells was calculated with the lowest Mann-Whitney U test p value (using a p value cut-off of < 0.01) (Tables S9A and S9B).
  ○ BMDC-TNF: Genome-wide CRISPR screen in mouse BMDC to identify genes that control the induction of tumor necrosis factor (Tnf) by bacterial lipopolysaccharide (LPS).^77^ Differential expression (DE) analysis of the genome-wide screen in “Tnflo” relative to “Tnfhi” was performed using DESeq2. Rank (column E) based on the p-value (include positive and negative regulators). Standard DESeq output (columns F–K), including mean expression of all guides targeting each gene (column F) and the fold change (column G) between “Tnflo” and “Tnfhi.” Positive values indicate enrichment in the “Tnflo” library and therefore positive regulators. Negative values indicate depletion in the “Tnflo” library and therefore negative regulators (Tables S10A and S10B).
  ○ TH2: Genome-wide CRISPR screen in TH2-differentiated T-cells that were isolated from transgenic mice expressing Cas9 and fluorescent reporters for Il4, Il13, Xbp1, and Gata3. LogFC are reported at gene level for guides enriched in high vs low populations at 72h post differentiation to TH2 phenotypes.^78^ Data were analyzed by BaIOPSE and MAGeCK, and reported at gene level as logFC, p-value, and ranked score (Tables S11A and S11B).
• *MGI Mouse Knockout Phenotypes* – Gene annotations for immunological and BrCa phenotypes from genetically engineered mouse models (GEMMs) were downloaded from the MGI database^112^ (Tables S12A and S12B).
• *COSMIC Cancer Gene Census* – Cancer driver gene annotations for BrCa were queried from Cancer Gene Census list in the COSMIC database^113^ (Table S13).
• *Leukocyte eQTL* – Genes annotated were annotated for significant eQTL (FDR <0.05) across 28 immune cell types from 416 donors in the ImmuNexUT database.^114^ Effect sizes for significant associations were used as inputs for the random forest model (Table S14).

#### LSP1 transcription expression in human breast cancer biopsies by scRNAseq analysis

The BioTuring BBrowser3 was used to visualize cell type clusters and LSP1 transcript expression from 14 treatment-naïve breast cancer patient scRNAseq dataset with a total of 44,024 cells profiled.^37^ Differential expression of LSP1 was performed using the BioTuring package “Venice”, as described previously.^80^ Briefly, LSP1 differential expression analysis was run comparing the T/NK cell population (14,395 cells) to the cancer cell population (16,235 cells) as defined by the BioTuring software cell-type specific markers and using the Venice method implemented in the BioTuring BBrowser3 interface.

#### Analysis of patient outcome associated with LSP1 protein expression

Clinical outcome data (progression-free survival; PFS, median follow-up 8.8 years) were available for the 1,109 ER+ BrCa patients. A data-driven optimal cutpoint for dichotomization (High vs. Low) of the LSP1 expression in CD45-positive cells was derived using X-tile^115^ and the prognostic value of the resulting dichotomized biomarker was evaluated using the Log-rank test and Kaplan-Meier curves (IBM SPSS).

#### RNAseq data analysis

Genome sequence and GTF files were obtained from Ensembl. The RSEM (RNA-Seq by Expectation-maximization) program function “rsem-prepare-reference” (v1.3.0) was used to extract the transcript sequences from the mouse genome (build GRCm38)^83^ and to generate Bowtie2 indices (Bowtie2 v2.2.8)^86^ followed by read alignment using the “rsem-calculate-expression” function. Differential expression analysis was performed using the Bioconductor package DESeq2 version 1.16.1^87^ to compute log2 fold-changes and FDR-adjusted *P* values. Molecular and functional pathway enrichment was measured using the Ingenuity Pathway Analysis (IPA) tool (QIAGEN, Redwood City, CA). The pathway activation scores were measured using the *Upstream Regulator Analysis* function of the Ingenuity Pathway Analysis (IPA) tool using the default settings (please see http://pages.ingenuity.com/rs/ingenuity/images/0812%20upstream_regulator_analysis_whitepaper.pdf) for a full description of the method. The upstream regulators in Figure 5A were unbiasedly selected based on three factors: (1) significant (FDR < 0.1) differential expression of the upstream regulator in E0771 tumors implanted in PWD.B6 mice compared with B6 homozygous mice, (2) evidence pathway changes (i.e., an observable pathway z-score), and (3) passing the accepted statistical threshold for the *Upstream Regulator Analysis* function (p < 0.01). Gene set enrichment analysis (GSEA) and enrichment of tumor infiltrate signatures was estimated using single-sample GSEA (ssGSEA) using a compiled list of immune 23 signatures,^88^^,^^116^^,^^117^^,^^118^^,^^119^^,^^120^ as implemented by the R package “GSVA” (v 1.34.0),^89^ and following ortholog mapping between human and mouse genes using the “biomaRT” R package.^84^

#### Mouse model linkage analysis

A single-locus scan was performed and LOD scores were calculated at 0.5 cM interval across the genome, using the imputation method implemented in R/qtl^81^^,^^82^ and significance was determined on the basis of 1,000 permutations of the data.^81^ A LOD score exceeding the 0.1 genome-wide adjusted threshold was considered significant.^121^ The Bayes credible interval function in R/qtl (bayesint) was used to approximate the 95% confidence intervals for the QTL peak location for both the additive and the interactive models.^122^ Mouse QTL boundaries were converted from cM to Mb using the Mouse Map Converter tool (http://cgd.jax.org/mousemapconverter/), and genomic features and variant effect predictions (VEP) were annotated using the “Genes and Markers Query” and “Search Mouse SNPs” functions of the Mouse Genome Informatics (MGI) database (http://www.informatics.jax.org/).

#### RT-qPCR analysis of MMTV-PyMT expression

Total RNA was extracted by Trizol from tumors extracted from homozygous B6-Tg(MMTV-PyMT) mice and heterozygous PWD.B6-Tg(MMTV-PyMT) F_1_ mice (n = 4 per group). cDNA was synthesized from 1 μg of total RNA using Advantage RT-cDNA kit (Clonetech, CA). Quantitative RT-PCR was performed using GoTaq qPCR Master Mix (Promega) on an ABI 7900 Real-Time. A typical reaction consisted of an initial denaturation step at 95°C for 5 minutes followed by 40 cycles of denaturation at 95°C for 15 seconds and annealing, extension, and data acquisition at 60°C for 1 minute. A final melting curve for each primer was calculated by heating from 60°C to 90°C. Data were normalized to r18s, and relative mRNA expression was determined using the ΔΔ*C*_*t*_ method: 2^-ΔΔCt^ = [(Ct gene of interest – Ct internal control) sample A) – (Ct gene of interest – Ct internal control) sample B)].

## Data Availability

• Code for the IO target score is available at: https://github.com/zdezsoabbv/IO-target-scoring. • Data generated during the study are available in the supplemental tables and in the github repository https://github.com/zdezsoabbv/IO-target-scoring. • Any additional information required to reanalyze the data reported in this paper is available from the lead contact upon request (bridget.rileygillis@abbvie.com).

## Supplemental information

Document S1. Figure S1

Table S1. Breast cancer susceptibility GWAS studies, related to Methods

Table S2. Human Breast Cancer GWAS candidate genes, related to Figure 1

Table S3. Colocalization analysis of 155 human breast cancer loci and autoimmunity risk loci (n = 4,076 from OpenTargets), related to Figure 1A

Table S4. Natural Language Processing (NLP) and ontology-based Text and Data Mining (TDM) of the Online Mendelian Inheritance in Man (OMIM) database, related to Methods

Table S5. Genome-wide gene essentiality scores across 40 human breast cancer cell lines (n = 10 ER+, n = 30 ER-) in the DepMap (downloaded 4Q19), related to Methods S5A) original score, S5B) p-value, S5C) z-score.

Table S6. Genome-wide CRISPRi/a screens of the modifiers of IFNg, TNF, or IL2 production in TCR-stimulated human T-cells (Schmidt et al.), related to Methods S6A) original, S6B) z-score.

Table S7. Genome-wide CRISPRx screen of CD8^+^ T cell expansion in response to TCR stimulation (Shifrut, related to Methods S7A) original, S7B) z-score.

Table S8. DrugZ ouput summary from genome-wide CRISPRx screens performed in mouse 4T1 and EMT6 breast carcinoma cell lines propagated in the presence or absence of CTLs (Lawson), related to Methods S8A) original, S8B) z-score.

Table S9. Genome-wide CRISPRx screen of LPS induced NFKB activity in an immortalized bone-marrow-derived macrophage (iBMDM)-Cas9 with an NF-KB-GFP transgenic reporter, related to Methods S9A) original, S9B) z-score.

Table S10. Genome-wide CRISPR screen in mouse BMDC to identify genes that control the induction of tumor necrosis factor (Tnf) by bacterial lipopolysaccharide (LPS), related to Methods S10A) original, S10B) z-score.

Table S11. Genome-wide CRISPR screen in TH2-differentiated T-cells that were isolated from transgenic mice expressing Cas9 and fluorescent reporters for Il4, Il13, Xbp1, and Gata3, related to Methods S11A) original, S11B) z-score.

Table S12. Mouse KO phenotypes related for the BCAC candidate genes downloaded from MGI, related to Methods S12A) immunity-related phenotype, S12B) mammary-related phenotype.

Table S13. COSMIC Cancer Census - annotated breast cancer genes, related to Methods

Table S14. Genes annotated were annotated for significant eQTL (FDR <0.05) across 28 immune cell types from 416 donors in the ImmuNexUT database, related to Methods

Table S15. Hierarchical clustering of BrCa candidates within the top 10% of I-O target probability scores, related to Methods S15A) Cluster1 enrichment analysis results, S15B) Cluster2 enrichment analysis results.

Table S16. Breast cancer GWAS candidate I-O target probability scores from random forest and elastic net models, related to Figure 1

Table S17. LSP1 Breast Cancer GWAS locus and LD block variants, related to Figure 2

Table S18. LSP1 protein level multivariable Cox proportional hazards regression analysis, related to Figure 2

Table S19. Differentially expressed genes from EO771.LMB tumors that were xenografted into B6 vs. PWD.B6 mice (FDR <0.1), related to Methods

Table S20. Pathway activation scores from EO771.LMB tumors that were xenografted into B6 and PWD.B6 mice, related to Figure 5A

Table S21. Mouse candidate genes localized within mouse chromosome 4 (4.68–55.84 Mb) and chromosome 5 (15.97–66.36 Mb) regions, related to Figure 4 S21A) 576 mouse candidate genes and annotation, S21B) Variant effect predictions (VEP) of SNPs located in candidate genes.
