# Supplementary material for: Machine learning reveals genetic modifiers of the immune microenvironment of cancer
Source: iScience. 2023 Aug 9;26(9):107576. doi: 10.1016/j.isci.2023.107576 (PMC10470213; doi:10.1016/j.isci.2023.107576)

iScience, Volume *26*

Supplemental information

Machine learning reveals genetic modifiers of the immune microenvironment of cancer

Bridget Riley-Gillis, Shirng-Wern Tsaih, Emily King, Sabrina Wollenhaupt, Jonas Reeb, Amy R. Peck, Kelsey Wackman, Angela Lemke, Hallgeir Rui, Zoltan Dezso, and Michael J. Flister

Figure S1. Comparison of I-O probability scores generated using random forest or elastic-net logistic regression, related to Figure 1.

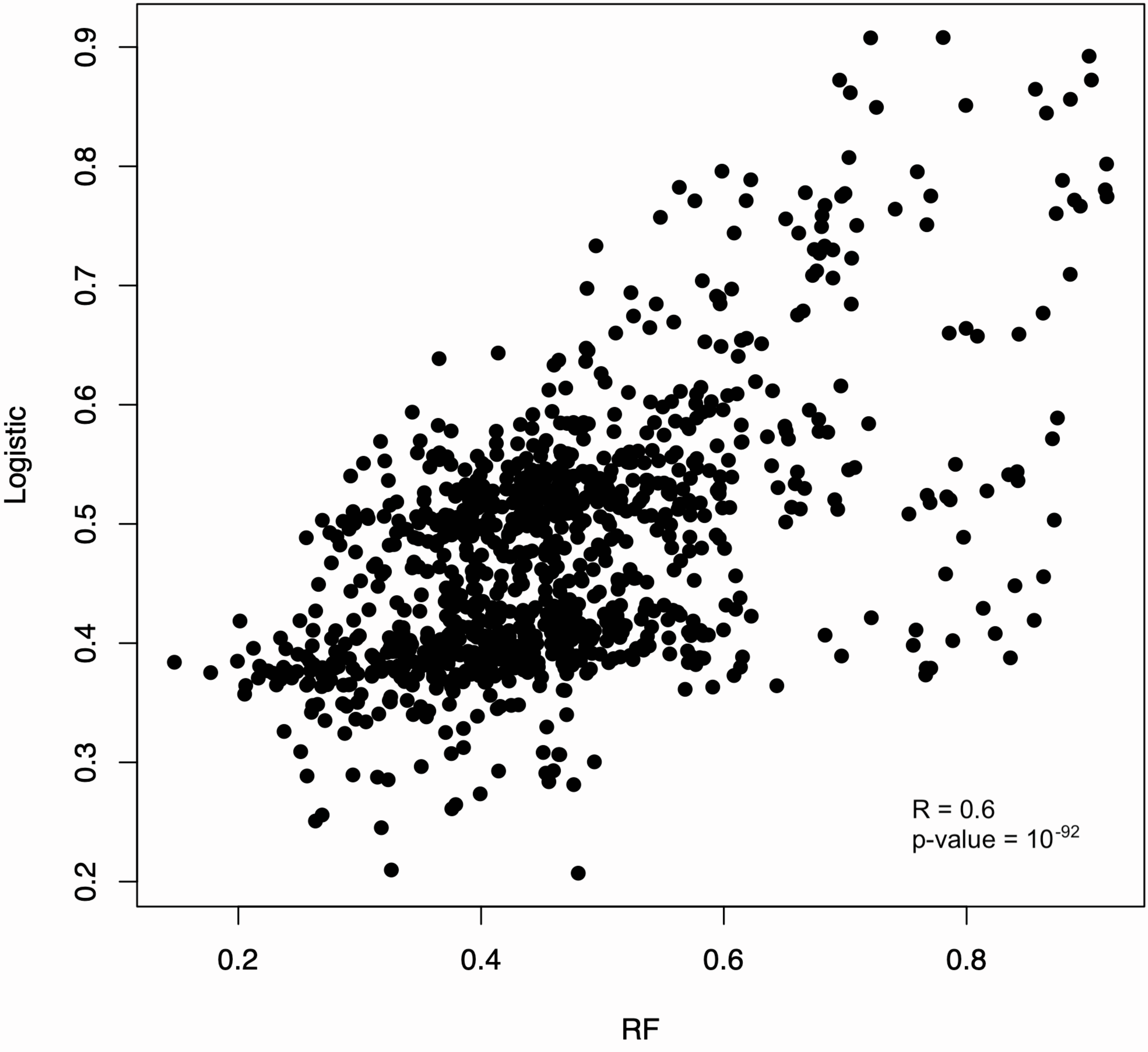

Supplement: Document S1. Figure S1 [file mmc1.pdf]
